# Hydrogels for Agricultural Applications: From Soil Amendment to Crop Enhancement

**DOI:** 10.3390/gels12050413

**Published:** 2026-05-09

**Authors:** Luohui Wang, Jihang Hu, Liyun Wang, Xiaobo Xue, Panrong Guo, Youming Dong, Fei Xiao, Cheng Li, Limin Guo

**Affiliations:** 1 College of Forestry, Henan Agricultural University, Zhengzhou 450002, China; 2 College of Materials Science and Engineering, Nanjing Forestry University, Nanjing 210037, China; 3 Research Institute of Wood Industry, Chinese Academy of Forestry, Beijing 100091, China; 4 Hunan Academy of Forestry, Changsha 410018, China

**Keywords:** hydrogel, soil amendment, water retention, crop growth, sustainable agriculture

## Abstract

Hydrogels (HGs), three-dimensional cross-linked hydrophilic polymer networks, have emerged as a promising class of functional materials for sustainable agriculture due to their exceptional water retention capacity, responsiveness to environmental stimuli, and favorable biocompatibility. This review systematically summarizes the key functional properties of hydrogels and critically examines their multidimensional roles within agricultural systems. The major synergistic benefits of hydrogels are highlighted, including (1) improvement of soil physical structure, chemical properties, and the biological microenvironment, thereby facilitating soil remediation; (2) direct enhancement of seed germination, root development, and crop productivity when employed as soil amendments or seed-coating materials; (3) controlled and sustained release of water, nutrients (N, P, K, and trace elements), and pesticides, leading to significant improvements in resource use efficiency; (4) functional delivery of beneficial microorganisms, enabling precise regulation of their activity and efficacy; and (5) advancement of soilless cultivation technologies through the development of sophisticated hydrogel-based substrates. Furthermore, this review discusses the key challenges that currently limit large-scale agricultural implementation, including insufficient biodegradability, potential ecotoxicological risks, and techno-economic constraints. Finally, future research directions are proposed from an interdisciplinary perspective, emphasizing rational material design, performance optimization, and practical field application. This comprehensive review aims to provide systematic theoretical guidance and practical insights for the development and deployment of hydrogel-based technologies in sustainable agriculture.

## 1. Introduction

Global agriculture is facing intensifying resource constraints and mounting environmental pressures. According to United Nations projections, the global population is expected to reach 9.7 billion by 2050, requiring an approximately 60% increase in food production compared with current levels [1]. Meanwhile, nearly 33% of the world’s arable land is severely degraded due to soil compaction, salinization, and organic matter depletion, resulting in sustained declines in agricultural productivity [2,3]. Agriculture accounts for nearly 70% of global freshwater withdrawals, yet water use efficiency remains generally low. Conventional high-input farming practices, characterized by annual pesticide consumption exceeding 1.9 million metric tons and irrigation water loss rates of up to 50%, aggravate soil pollution and ecological imbalance, reinforcing a vicious cycle of “increased yield demand–resource scarcity–environmental cost” [4]. Consequently, the development of innovative technologies that can simultaneously advance the United Nations Sustainable Development Goals (SDGs), particularly “Zero Hunger” (SDG 2) and “Life on Land” (SDG 15), has become increasingly urgent [5].

In this context, hydrogels (HGs), defined by their unique three-dimensional hydrophilic polymer networks, have attracted growing attention as a versatile functional material for addressing agricultural sustainability challenges. Their capacity to absorb and retain water, often hundreds to thousands of times their own weight, enables effective improvement of soil moisture availability and physical structure [6]. More importantly, the responsiveness of hydrogels to environmental stimuli such as pH, temperature, and ionic strength allows for the controlled and on-demand release of water, nutrients, and agrochemicals. In addition, hydrogels can function as carriers for beneficial microorganisms and as remediation agents for saline–alkali soils, thereby synergistically enhancing soil health and fertility [7,8,9]. Although early hydrogel research, dating back to the 1980s, was largely concentrated in sanitary and biomedical fields, the past two decades have witnessed rapid expansion in both fundamental studies and agricultural applications. These now encompass soil conditioning, water-saving irrigation, fertilizer use efficiency enhancement, seed engineering, and agricultural pollution remediation [10,11,12]. Despite these advances, the large-scale implementation of hydrogel technologies in agriculture remains constrained by multidimensional bottlenecks related to material performance, environmental compatibility, and economic feasibility. Synthetic hydrogels, such as polyacrylamide-based systems, often exhibit slow degradation rates and raise concerns regarding potential ecotoxicity. Conversely, hydrogels derived from natural polymers (e.g., chitosan and cellulose) typically suffer from insufficient mechanical strength and limited environmental stability, restricting their long-term field performance [13,14].

Against this backdrop, this review focuses on the core action pathway of hydrogels, spanning from soil property modification to crop growth promotion, and systematically summarizes their multilevel mechanisms and recent advances in agricultural systems. It integrates perspectives from materials science, agronomy, and environmental science, addressing hydrogel design principles, agricultural efficacy, environmental risks, and future development directions. This review encompasses the primary application pathways of hydrogels in agriculture, focusing on soil amendment and seed coating as direct delivery methods to the rhizosphere, while also discussing their emerging roles in advanced soilless cultivation systems. Foliar applications, while a potential niche, are beyond the scope of this work due to distinct formulation and delivery challenges. Specifically, this review centers on two key questions: (1) how hydrogels optimize the rhizosphere microenvironment through regulation of soil physical, chemical, and biological processes; and (2) how hydrogels enhance crop productivity and resource use efficiency via intelligent water and nutrient management. Unlike reviews that primarily catalog material types or isolated functions, this work emphasizes the systemic “soil-to-plant” action pathway and critically examines the practical bottlenecks that hinder field-scale translation, such as performance durability under cyclic conditions and compatibility with agricultural operations. Ultimately, this work aims to provide researchers, agricultural practitioners, and policymakers with a comprehensive knowledge framework and practical reference to guide the efficient, safe, and sustainable application of hydrogels in modern agriculture.

To provide a clearer overview, the main categories of hydrogels relevant to agriculture are summarized in Table 1, based on their origin, cross-linking mechanisms, and functional characteristics.

### Literature Search Strategy and Scope

To ensure a comprehensive and unbiased coverage of recent advances, a systematic literature search was conducted up to March 2026. The primary databases included Web of Science, Scopus, and Google Scholar. The search utilized a combination of keywords: (“hydrogel” OR “superabsorbent polymer”) AND (“agriculture” OR “soil” OR “crop” OR “water retention” OR “fertilizer release” OR “seed coating”). The initial search results were screened based on titles and abstracts, focusing on peer-reviewed research articles, reviews, and significant conference proceedings published predominantly within the last decade. Studies were selected for in-depth discussion if they provided clear insights into (1) novel hydrogel material design relevant to agriculture, (2) mechanistic understanding of hydrogel–soil–plant interactions, or (3) field-scale validation of hydrogel efficacy. Emphasis was placed on work that bridges material science with agronomic and environmental outcomes.

## 2. Impacts of Hydrogels on Soil Physicochemical Properties

### 2.1. Soil Bulk Density and Porosity

Hydrogels modify soil structure through dual mechanisms of physical expansion and interfacial cementation: (1) Expansion Effect: Upon water absorption, hydrogels can swell hundreds of times in volume (typically 200–1000 fold), physically pushing apart soil particles and reducing bulk density. (2) Cementation Effect: Polymer chains interact with soil clay particles via hydrogen bonding, ionic bonds, etc., promoting the formation and stabilization of soil aggregates. Additionally, hydrogels optimize pore architecture by increasing non-capillary macropores beneficial for aeration, acting as “bridges” between particles to improve pore connectivity, thereby enhancing hydraulic conductivity and aeration, and fostering the formation of more water-stable aggregates in the 0–2 mm size range. Studies indicate that hydrogel treatment can increase the proportion of water-stable aggregates by over 40%, effectively mitigating soil crusting and erosion risks. Crucially, the soil structural improvements conferred by hydrogels are not permanent and can diminish over multiple wet–dry cycles. The repeated swelling and shrinkage exert mechanical stress on the polymer network, potentially leading to fatigue, fragmentation, or chemical degradation over time. Synthetic hydrogels typically exhibit higher resilience to swelling cycles [15]. In contrast, natural polymer-based hydrogels may suffer from faster mechanical and chemical degradation. This contrast reflects the inherent trade-off between persistence and biodegradability. Synthetic hydrogels provide durable soil amendment but risk accumulating as persistent fragments, raising concerns about long-term soil health and microplastic generation. Conversely, natural hydrogels degrade rapidly and are environmentally benign, yet often require seasonal re-application, reducing economic feasibility.

The efficacy of hydrogels in amending soil bulk density and porosity is predominantly influenced by soil texture and application rate, with soil texture being the key determinant. The most pronounced improvements are typically observed in structurally loose, initially high-porosity sandy soils. For example, Malik et al. [16] reported that a 0.3% application of carboxymethyl tamarind kernel gum-poly(sodium methacrylate)-polyacrylamide composite hydrogel reduced the bulk density of sandy soil by 13% (1.9 g/cm^3^) and increased total porosity by 29%. In contrast, for clay soil, the reductions in bulk density and increases in total porosity were only 1.7% (1.19 g/cm^3^) and 5.9%, respectively, primarily attributed to the limited space for physical expansion between dense clay particles. Similarly, Errahali et al. [17] found that a 0.5% application of a polyacrylamide-potassium acrylate superabsorbent polymer (SAP) decreased the bulk density of sandy loam by 54.54% (0.88 g/cm^3^), with an even greater reduction of 56.98% (0.93 g/cm^3^) observed in clay loam. From the perspective of material type, hydrogels—whether synthetic, natural-based, or composite—all demonstrate potential for improving soil physical structure. For instance, phosphorus-doped okara-derived hydrogel (POKgel) developed by Jausoro et al. [18] effectively reduced soil bulk density (1.112 g/cm^3^) and increased porosity (55%), thereby improving soil aeration and water/nutrient supply. The carboxymethyl cellulose-g-poly(acrylic acid-co-acrylamide)/diatomite/diammonium phosphate (DAP) drug-loaded composite hydrogel fabricated by Barala et al. [19] also improved soil bulk density, porosity, organic carbon content, and microbial activity. Research by Malik et al. [20] showed that a 0.5% CMTKG-PSMA hydrogel treatment increased total soil porosity by 14% and reduced bulk density by 8%. The study by Nascimento et al. [21] further confirmed that polyacrylamide-potassium acrylate hydrogel consistently outperformed untreated controls in improving soil bulk density and porosity across different temperatures and treatment durations, demonstrating good environmental stability.

Notably, the application rate of hydrogels generally exhibits a positive dose–response relationship, with higher concentrations often leading to more significant improvements. A comparative study by Solieman et al. [22] found that in sandy and calcareous loam soils, a 0.4% application rate of bio-based straw hydrogel (SH) and acrylamide hydrogel (AH) outperformed a 0.2% rate in improving soil hydro-physical, physicochemical, and biological properties. Specifically, the higher concentration (0.4%) of SH showed superior performance in enhancing soil biological characteristics, while 0.4% AH had a slight advantage in short-term (5–6 days post-irrigation) water retention and hydro-physical property improvement.

### 2.2. Soil Water Retention

Hydrogels (HGs) substantially enhance soil water-holding capacity (WHC) and water retention capacity (WRC) by modifying soil pore structure and interfacial properties. Water-Holding Capacity (WHC) refers to the maximum amount of water that a soil (or soil–hydrogel system) can retain against gravity after saturation and drainage, often approximated by field capacity (FC) [23]. WHC represents the static water storage potential of the system. Water Retention Capacity (WRC), often described by the soil water retention curve, characterizes the relationship between soil water content and matric potential (suction) [24]. It encompasses the dynamic ability of the soil to retain water across a range of moisture tensions, from saturation to the wilting point. In the context of hydrogel amendment, hydrogels contribute to both WHC and WRC: their primary function lies in expanding the range of plant-available water (PAW), primarily through increasing field capacity (FC) and, in some cases, lowering the wilting point (WP). This enhancement ensures a more sufficient and stable water supply for plant growth. However, conventional synthetic water-retaining agents (WRAs), such as potassium polyacrylate, suffer from poor biodegradability and potential environmental risks, while natural polymer-based hydrogels typically exhibit relatively low water absorption capacity (WAC), often below 500 g/g. Consequently, recent research has focused on the development of advanced hydrogel materials that integrate high water absorption performance with improved environmental compatibility. Major progress in this field has been achieved through the innovative design of the following material categories.

**Figure 1:**
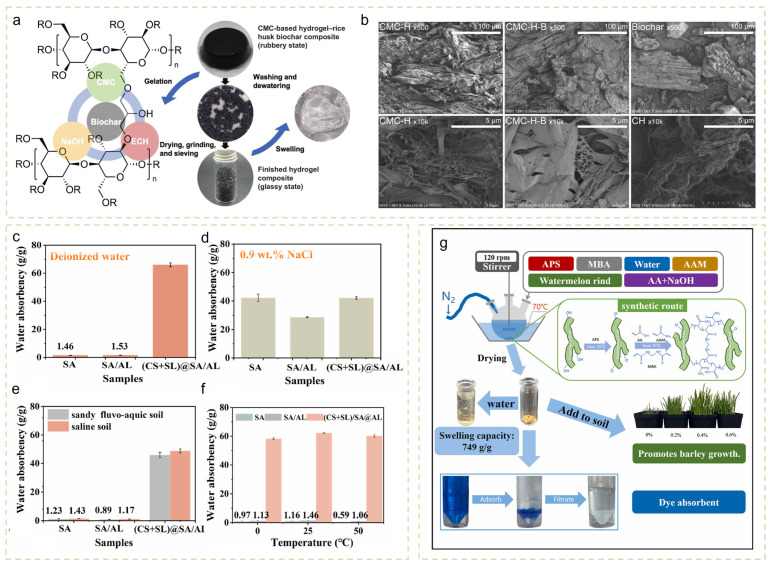
(**a**) Schematic illustration of CMC-H-B hydrogel synthesis. (**b**) Field-emission scanning electron microscopy (FESEM) images of CMC-H-B hydrogel and its biochar composite, revealing the internal porous network structure. Reproduced with permission from reference [27]. Copyright 2025, Elsevier. (**c**–**f**) Water absorption performance of (CS + SL)@SA/AL hydrogel in different media: (**c**) deionized water, (**d**) NaCl solution, (**e**) soil leachate, and (**f**) variations in water absorption capacity at different temperatures. Reproduced with permission from reference [28]. Copyright 2025, Elsevier. (**g**) Schematic of the preparation process of watermelon rind-based WR hydrogel and its application in soil water retention. Reproduced with permission from reference [30]. Copyright 2024, Elsevier.

(1) Superabsorbent Natural Composite Hydrogels. Through compositing strategies and structural optimization, substantial improvements in the water absorption performance of natural-based hydrogels have been achieved. Barala et al. [25] developed a diammonium phosphate-composite hydrogel that increased soil water retention by 285% at an application rate of 0.3%. Liu et al. [26] reported that a 0.5 wt% fenugreek gum-borax hydrogel increased the expansion index of sandy soil from 16.28% to 35.53% and extended the water retention duration from 2 days to 11.5 days under conditions of 20 °C and 60% relative humidity. Ye et al. [27] fabricated a fully biomass-based hydrogel-biochar composite (CMC-H-B). Owing to its loose three-dimensional network structure formed by low crosslinking density, this composite achieved an exceptionally high WAC of 3240.07 g/g (Figure 1a,b). Soil application experiments demonstrated that the addition of only 0.1 *w*/*w*% CMC-H-B increased the WHC of sandy loam by 91.67%.

(2) Salt-Alkali-Tolerant Functionalized Hydrogels. To mitigate ionic stress in saline-alkali soils, hydrogel functionalization has emerged as a critical strategy. Li et al. [28] developed a zwitterionic (CS + SL)@SA/AL hydrogel, whose WAC in high-salt alkaline environments reached 34–42 times that of conventional calcium alginate hydrogel, with values up to 69.33 g/g (Figure 1c–f). Xiong et al. [29] prepared a composite hydrogel via in situ polymerization of hydroxyethyl starch (HES) and 2-acrylamido-2-methyl-1-propanesulfonic acid (AMPS) in the presence of inorganic clay, using calcium alginate (CA33) as a regulation carrier. This material reached water absorption equilibrium within 30 min, exhibiting WAC values of 1484 g/g in distilled water, 312 g/g in tap water, and 121 g/g in 0.9% saline solution. The incorporation of calcium alginate further enhanced water retention and slow-release performance by 25.8%. These functionalized hydrogels offer effective solutions for water management in high-salinity soils and saline-alkali land reclamation.

(3) Agricultural Waste-Based Hydrogels. The Utilization of agricultural waste as feedstock for hydrogel synthesis represents an important strategy for resource and cost reduction. Teng et al. [30] fabricated a hydrogel from watermelon rind with a WAC of 749 ± 32 g/g, retaining 94.88% of its absorption capacity after 8 absorption–drying cycles and significantly enhancing water retention across multiple soil types (Figure 1g). Chen et al. [31] synthesized an environmentally friendly hydrogel based on a locust bean gum-borax system, achieving a WAC of 130.29 g/g. The incorporation of 0.9% hydrogel increased the maximum water content of sandy soil by 32.03%, extended water retention time to 14 days, and simultaneously increased soil organic matter content by 8.64 g/kg, demonstrating favorable soil amendment effects with low environmental risk.

(4) Lignin- and Cellulose-Based Hydrogels. Lignin and cellulose, as abundant and renewable biomass resources, are well suited for the development of environmentally benign hydrogels. The lignin-based hydrogel synthesized by Adjuik et al. [32] exhibited WAC values of 2013%, 1092%, and 825% in deionized water, tap water, and 0.9% NaCl solution, respectively, and application rates of 0.3–1% effectively improved water retention in silt loam soil. Kadry et al. [33] prepared a cellulose-based hydrogel extracted from rice straw, which showed remarkably high WAC values of 9811% in tap water and 3121% in hard water, while retaining 64.58 wt% of absorbed water after 4 days of outdoor exposure. Guo et al. [34] fabricated a cellulose-based superabsorbent hydrogel via chemical grafting, exhibiting water absorption rates of 604% in distilled water and 119% in saline solution, highlighting its potential for mitigating soil salinization and drought stress.

### 2.3. Soil pH, Electrical Conductivity (EC), and Cation Exchange Capacity (CEC)

Hydrogels can effectively regulate key soil chemical properties—pH, electrical conductivity (EC), and cation exchange capacity (CEC)—through the dissociation of active functional groups within their polymer networks, ion exchange, and buffering effects. The underlying mechanisms include: (1) pH regulation, whereby hydrogels containing functional groups such as carboxyl (-COOH) or amino (-NH_2_) moieties buffer soil acidity or alkalinity via proton exchange; pH-responsive hydrogels can further function as intelligent, stimulus-responsive carriers. (2) EC regulation, in which hydrogels adsorb or immobilize free salt ions (e.g., Na^+^, Cl^−^), thereby directly reducing soil EC and alleviating salt stress. (3) CEC enhancement, achieved by introducing additional negatively charged adsorption sites that strengthen the soil’s nutrient-holding capacity.

**Figure 2:**
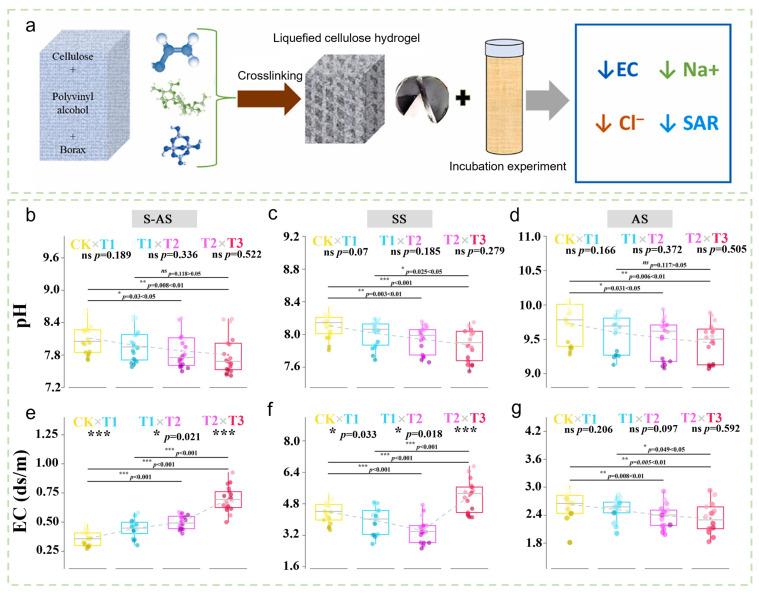
(**a**) Co-cultivation process of liquefied sugarcane cellulose hydrogel with soil. Reproduced with permission from reference [35]. Copyright 2022, Elsevier. (**b**–**d**) Comparison of soil pH under different CWR-SRMs hydrogel treatment gradients. (**e**–**g**) Comparison of soil electrical conductivity (EC) under different CWR-SRMs hydrogel treatment gradients. Reproduced with permission from reference [36]. Copyright 2024, Elsevier. * *p* < 0.05, ** *p* < 0.01, *** *p* < 0.001.

In the remediation of saline-alkali soils, hydrogels have demonstrated considerable potential for desalination and ion adsorption. B. Tefera et al. [35] applied a sugarcane bagasse/PVA/borax hydrogel at a 3% rate, which significantly reduced the EC of pore water in saline soil, as well as the concentrations of ions such as Cl^−^ and Ca^2+^, although this was accompanied by an increase in soil pH (Figure 2a). Qi et al. [36] developed CWR-SRMs hydrogels that, through their ion-adsorption network, simultaneously reduced soil pH (12.3–14.1%), EC, exchangeable sodium (E(Na)) (38.59–47.73%), and sodium adsorption ratio (ESP) in saline-alkali soils. Moderate application rates also increased soil CEC and the content of key nutrients, including nitrogen, phosphorus, and potassium, while enhancing soil water retention (Figure 2b–g). Abdeen et al. [37] further confirmed that poly(vinyl alcohol) borate hydrogel could selectively adsorb excess Na^+^ from saline beach soil, effectively reducing both EC and sodium content. Notably, the direction and magnitude of these effects depend strongly on hydrogel composition. For example, the poly(acrylic acid-co-acrylamide)/potassium humate superabsorbent hydrogel nanocomposite (PHNC) developed by Shahid et al. [38] reduced soil pH by 2–5% at application rates of 0.1–0.4%; however, due to the release of potassium ions from humate, soil EC increased by 6–57%.

Beyond salinity regulation, hydrogels also exhibit synergistic effects in nutrient retention and soil fertility improvement. Zhou et al. [39] reported that the LR-g-PAA/MMT/urea composite hydrogel significantly increased soil organic matter and nitrogen and phosphorus reserves by enhancing the CEC (7.66 cmol(+)/kg). Souza et al. [40] demonstrated that combining cellulose-based hydrogels with arbuscular mycorrhizal fungi resulted in marked increases in soil CEC (45.89 cmol(+)/kg) and organic carbon content, highlighting the potential for integrated physico-chemical–biological soil improvement strategies. Furthermore, a 9-year field trial conducted by Lentz et al. [41] showed that long-term application of polyacrylamide or polyacrylate hydrogels sustainably improved soil pH, EC, and organic carbon content in degraded soils, although attention should be paid to potential nutrient balance issues, such as reduced magnesium availability.

With respect to material formulation, Jong et al. [42] systematically investigated the influence of different plasticizers on the performance of cellulose-based hydrogel. They found that a hydrogel composed of sodium carboxymethyl cellulose (NaCMC, 1.75% *w*/*w*), glycerol (1.0% *w*/*w*), and polyethylene glycol (PEG, 0.5% *w*/*w*) exhibited optimal properties, with a water absorption rate exceeding 2600% and the ability to retain at least 10% moisture for 96 h. Soil application experiments demonstrated that this hydrogel effectively increased soil moisture content, pH, and EC across four different soil types (sand, topsoil, mud, and clay) over 15 days.

### 2.4. Soil Remediation and Structural Enhancement

Hydrogels exhibit multifaceted application potential in soil remediation and structural reinforcement. Their primary mechanisms can be summarized as follows: (1) Physical barrier formation and microenvironment modulation, whereby repeated swelling and shrinkage enable hydrogels to form a protective layer on the soil surface, effectively reducing water evaporation and nutrient leaching. (2) Aggregate formation and erosion resistance, in which hydrogels act as binding agents that promote the formation of stable, water-resistant soil aggregates, thereby markedly enhancing resistance to water and wind erosion, particularly in structurally loose sandy soils or sloping agricultural land. (3) Pollutant adsorption and immobilization, wherein functionalized hydrogels selectively adsorb contaminants such as heavy metals, reducing their bioavailability and mobility within the soil matrix.

**Figure 3:**
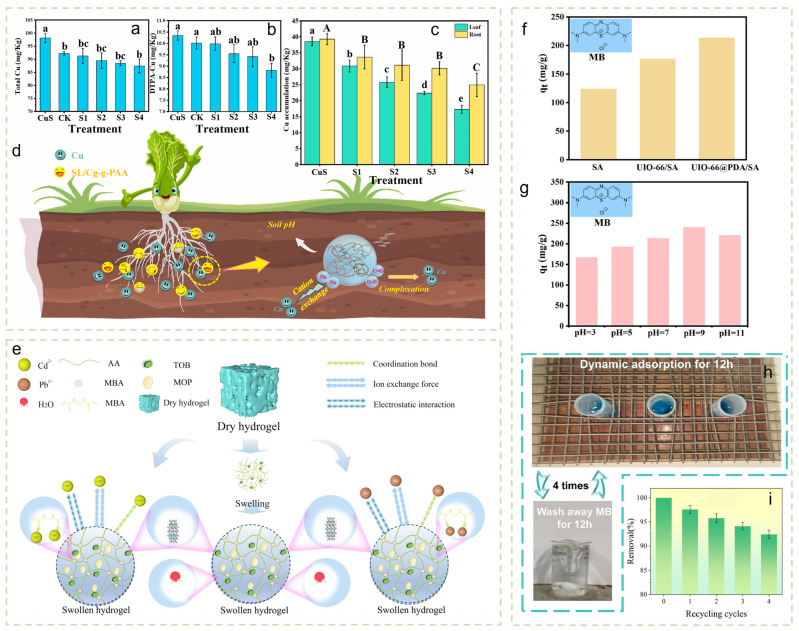
(**a**) Total copper content in soil per treatment with SL/Cg-g-PAA hydrogel. (**b**) DTPA-extractable Cu content in soil per treatment. (**c**) Copper content in leaves and roots of pak choi per treatment. (**d**) Passivation mechanism of copper in soil by SL/Cg-g-PAA hydrogel. Reproduced with permission from reference [43]. Copyright 2024, Elsevier. (**e**) Schematic diagram of the Cd/Pb removal and water absorption mechanism of MxTy-CH hydrogel. Reproduced with permission from reference [45]. Copyright 2024, Elsevier. (**f**) Adsorption capacity of UIO-66@PDA/SA hydrogel compared to SA and UIO-66/SA for MB. (**g**) Adsorption capacity for MB under different pH conditions. (**h**) Dynamic adsorption process. (**i**) Adsorption efficiency over 4 cycles. Reproduced with permission from reference [49]. Copyright 2024, Elsevier.

In the remediation of heavy-metal-contaminated soils, functionalized hydrogels have demonstrated high adsorption efficiency. Ding et al. [43] synthesized a sodium lignosulfonate/carboxymethyl cellulose-g-poly(acrylic acid) hydrogel, which reduced the available copper content in soil by 14.1% and decreased copper uptake in pak choi by 36–55% (Figure 3a–c), with the corresponding adsorption/passivation mechanism illustrated in Figure 3d. Li et al. [44] prepared an HLDT hydrogel modified with L-Dopa and tannic acid, exhibiting adsorption capacities of 193.3, 316.9, and 49.6 mM/g for Cd, Fe, and Pb, respectively; He et al. [45] reported a mandarin orange peel-based hydrogel with a high water absorption capacity of 204.29 g/g, alongside excellent adsorption capacities for Pb^2+^ (1743 mg/g) and Cd^2+^ (340 mg/g). The mechanisms governing Cd/Pb removal and water absorption are shown in Figure 3e. Furthermore, Ma et al. [46] demonstrated that their developed L-PH hydrogel inhibited the leaching of Cd^2+^, Cu^2+^, and Zn^2+^ by up to 83.6%, while simultaneously increasing soil organic carbon content and improving soil water retention.

Hydrogels have also shown notable effectiveness in saline-alkali soil improvement and structural enhancement. Wu et al. [47] developed a pH-sensitive semi-interpenetrating network (semi-IPN) hydrogel that sustained nutrient release for more than 30 days in saline-alkali soil, while concurrently increasing soil enzyme activity, enhancing available nutrient content, and lowering soil pH, ultimately improving wheat salt tolerance. In terms of soil structure stabilization and water–soil conservation, Liao et al. [48] prepared a PVA/SA interpenetrating network hydrogel via freeze–thaw cycling, which maintained soil aggregate stability above 99% after 70 days, increased soil cohesion to 80 kPa, and substantially reduced saturated hydraulic conductivity. Lu et al. [49] designed a core–shell-structured UIO-66@PDA/SA hydrogel composite bead that effectively retained soil moisture (evaporation rate < 1% within 4 h), exhibited a high adsorption capacity for methylene blue (213.79 mg/g), and maintained stable performance over 4 reuse cycles (Figure 3f–i).

## 3. Applications of Hydrogels in Crop Growth and Production

The promising results of hydrogels in crop enhancement, as discussed below, are predominantly derived from controlled laboratory and greenhouse pot studies. While these experiments provide invaluable mechanistic insights and proof of concept, their outcomes often magnify benefits compared to field conditions. The translation from pot to field involves scaling effects, uncontrolled environmental variables, soil heterogeneity, and interactions with farm management practices, all of which can significantly modulate hydrogel efficacy. Therefore, while the following sections summarize the state of the art, a critical perspective acknowledges that large-scale field validation remains the ultimate test for these technologies.

### 3.1. Hydrogels as Soil Amendments

**Figure 4:**
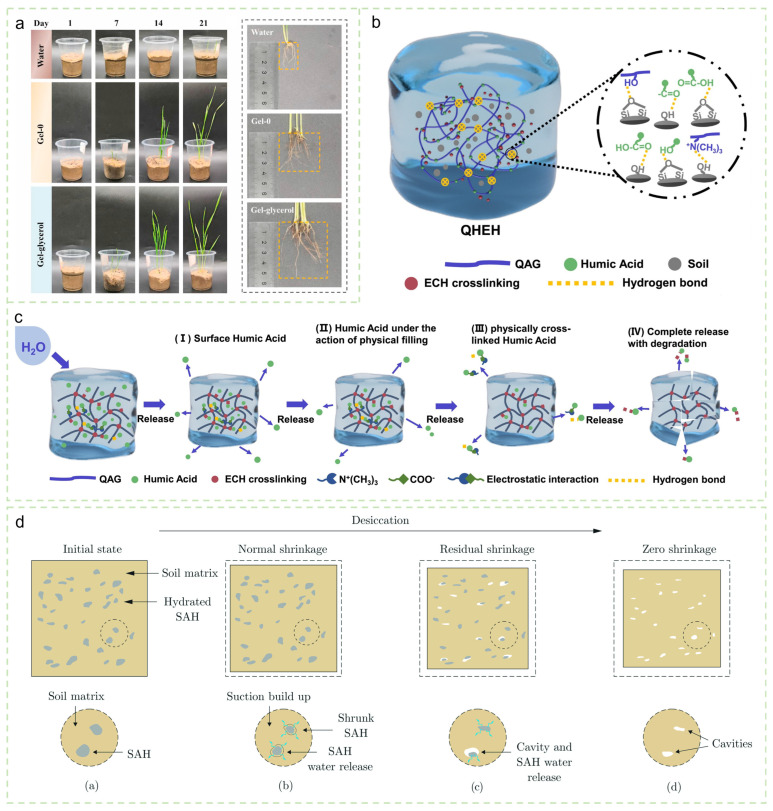
(**a**) Photographs of wheat plants grown in soil treated with water, Gel-0, and Gel-glycerol from day 1 to day 21, showing roots after 21 days of soil treatment. Reproduced with permission from reference [52]. Copyright 2022, Elsevier. (**b**) Schematic diagram of interactions between soil and QHEH hydrogel. (**c**) Schematic of HA release mechanism. Reproduced with permission from reference [56]. Copyright 2024, Elsevier. (**d**) Schematic of the interaction between soil matrix and SAH particles during the drying process. Reproduced with permission from reference [62]. Copyright 2024, Elsevier.

Hydrogels improve rhizosphere water status primarily through their ultra-high water-holding capacity. However, the potential environmental risks associated with conventional synthetic superabsorbent polymers (SAPs) cannot be overlooked. For example, Chen et al. [50] reported that high application rates of traditional SAPs inhibited plant growth, as residual acrylic acid monomers may interfere with plant metabolic processes, while excess sodium ions could exacerbate osmotic stress. To address these limitations, recent research has increasingly focused on the development of safer and more efficient hydrogel systems. Wang et al. [51] prepared a chitosan-carboxymethyl cellulose-silk fibroin (CS-CMC-SF) hydrogel via physical cross-linking, thereby avoiding residues from chemical cross-linkers. This material increased the water-holding capacity of saline-alkali soil by 103% and enhanced wheat root length by 38%. Qin et al. [52] developed a cellulose-glycerol-based “gel-glycerol” hydrogel with excellent thermal stability and a water retention capacity as high as 160%. After 21 days of application, wheat germination rate and average leaf number increased by 21.88% and 100%, respectively (Figure 4a). Ren et al. [53] designed an alginate/chitosan fumigant-loaded hydrogel capable of pH-responsive controlled release, which enhanced pest and disease control efficacy while substantially reducing chemical exposure risk.

As controlled-release nutrient carriers, hydrogels can effectively improve fertilizer use efficiency. Niu et al. [54] prepared an alginate-based hydrogel using urea as a cross-linker agent, enabling the sustained release of N, P, and K over a period of 9 weeks. Etminani-Esfahani et al. [55] developed a cellulose-based slow-release fertilizer that achieved a urea release rate of 61.5% over 42 days and simultaneously increased chlorophyll content in wheat leaves. Cui et al. [56] fabricated a quaternary ammonium guar gum/humic acid hydrogel, which exhibited dynamic bonding delayed humic acid release to only 35% over 240 h (Figure 4c). This hydrogel doubled soil water retention capacity and achieved a 100% mung bean germination rate. In addition, interactions between the hydrogel and soil minerals promoted hydrogel–soil and soil–carbonate adhesion (Figure 4b), increasing the adhesion strength by 650%. Iqbal et al. [57] demonstrated the valorization of waste materials by developing a wood ash/nanocellulose composite hydrogel, which significantly enhanced pea plant growth (37 leaves and 20.2 cm stem height), integrating water retention–nutrient supply functions and serving as an effective agricultural soil conditioner.

Hydrogels also exhibit multifunctionality in mitigating adverse soil conditions. Pettinelli et al. [58] reported that a sodium alginate-g-polyacrylamide hydrogel significantly increased the survival rate and photosynthetic efficiency of eucalyptus seedlings under drought stress. Albalasmeh et al. [59] confirmed that the addition of 0.33% hydrogel increased soil–plant-available water content by 49% and improved water use efficiency of corn to 41% and 67% in sandy and silt clay loam soils, respectively. Dai et al. [60] used waste leaves to prepare a sodium alginate/chitosan hydrogel, and a 5 wt% application increased seed germination by 40% and promoted root development under drought conditions. Cao et al. [61] prepared an activated carbon-agarose (Agar-AC) hydrogel, which enhanced canola seed germination and growth indices through the incorporation of activated carbon. Furthermore, hydrogels show considerable potential in soil structure reinforcement and surface protection. Liaudat et al. [62] demonstrated that the addition of 0.1% superabsorbent hydrogel (SAH) prolonged the normal soil shrinkage phase, effectively delaying crack formation during drying (interaction illustrated in Figure 4d). Li et al. [63] developed a self-healing chitosan-carbon dot hydrogel (COCu-K) that formed a continuous film on the soil surface, inhibiting 90% of weed seed germination while promoting chili seedling growth and achieving a degradation rate of 63.61% within 8 weeks.

Beyond water and macronutrient management, hydrogel soil amendments are increasingly engineered as delivery vehicles for plant growth regulators (PGRs) such as gibberellic acid (GA) [64]. Conventional foliar GA application is limited by rapid photodegradation, volatility, and uneven distribution. Encapsulation within hydrogel matrices provides an innovative soil-based delivery alternative that protects the bioactive compound and enables sustained, root-targeted release. Using an iono-tropic pre-gelation method, Bhat et al. [65] developed alginate–chitosan hydrogel beads incorporating diatomite for the extended release of GA. The addition of dopamine-functionalized nanoporous diatomite (DE–PDA) reinforced the hydrogel network via enhanced hydrogen bonding and ionic interactions, effectively modulating GA release kinetics and achieving a cumulative release of 86.3% by day 15. This hydrogel-based delivery system significantly outperformed direct GA application in enhancing tomato seed germination, achieving a 100% germination rate and promoting subsequent seedling growth. The study underscores the potential of hydrogels as intelligent carriers for phytohormones, offering a novel strategy for the precise regulation of plant development and stress resilience through the rhizosphere.

### 3.2. Hydrogel Seed Coatings for Enhanced Germination

Hydrogel seed coatings function by forming a physical barrier around seeds and regulate the immediate microenvironment, thereby effectively enhancing seed germination rate and seedling uniformity under abiotic stresses such as drought and salinity. The core functions of hydrogel seed coatings include: (1) sustained water release, in which the coating layer absorbs and gradually releases water, creating a localized moist microzone around the seed and prolonging water availability during germination; (2) functional substance delivery, whereby the hydrogel matrix serves as a carrier for fertilizers, biostimulants, or pesticides, enabling precise, and controlled release; and (3) microbial protection and colonization, as the porous hydrogel network provides shelter and colonization space for beneficial microorganisms. The primary role of hydrogel seed coatings is to ensure an adequate and stable water supply during seed germination. Pathak et al. [66] demonstrated that modified starch-based hydrogel coatings significantly improved maize emergence under limited water supply conditions (77% of crop water requirement). However, their direct influence on plant water availability diminished after seedling root establishment, underscoring the critical role of hydrogel coatings during the germination-to-early establishment phase. Under drought stress conditions, Tursynova et al. [67] developed a galangal gum-based hydrogel enriched with growth stimulants; wheat seeds coated with two layers of this hydrogel survived up to 10 days longer than uncoated controls under water deficit, indicating enhanced drought tolerance.

**Figure 5:**
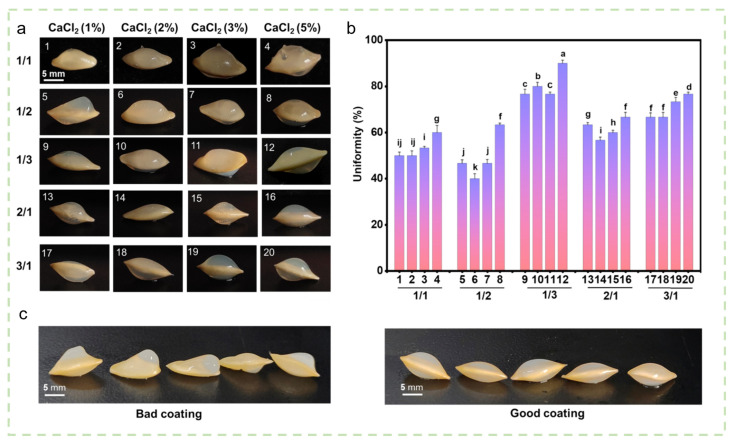
Morphological analysis of cucumber seeds coated with sodium alginate/pectin hydrogel film. (**a**) Representative images of cucumber seeds coated with SA/PC hydrogel at different SA/PC ratios and CaCl_2_ concentrations. (**b**) Uniformity of hydrogel coating on SA/PC-coated cucumber seeds. (**c**) Representative images of superior and inferior coatings on SA/PC hydrogel-coated cucumber seeds. Reproduced with permission from reference [70]. Copyright 2024, Elsevier.

Beyond water regulation, hydrogel coatings act as intelligent carriers for the sustained and targeted delivery of nutrients. Skrzypczak et al. [68] prepared a sodium alginate/NPK hydrogel coating that enabled gradual nutrient release, resulting in a 50% increase in cucumber seedling fresh weight and a fourfold increase in root length. Kumari et al. [69] developed a montmorillonite/starch/zinc composite coating that effectively prolonged zinc release, alleviating zinc deficiency in nutrient-poor soils and significantly improving seed germination rate and subsequent vegetative growth. In the context of biocontrol and microbial inoculation, the protective function of hydrogels coatings is particularly important. Abdukerim et al. [70] encapsulated the biocontrol bacterium *Bacillus subtilis* ZF71 within a sodium alginate/pectin hydrogel for cucumber seed coating, forming a biofilm-like structure with high viable cell counts on the seed surface (representative images in Figure 5a,c). High bacterial viability was maintained after 37 days of storage, and pot experiments demonstrated a control efficacy of 53.26% against *Fusarium* root rot, significantly outperforming conventional liquid bacterial irrigation. Moreover, the optimized SA/PC hydrogel formulation achieved a maximum coating uniformity of 90% on cucumber seeds (Figure 5b).

To address environmental variability, stimuli-responsive hydrogels enable the on-demand release of functional substances. Gao et al. [71] and Guan et al. [72] independently developed thermoresponsive hydrogel coatings based on poly(N-isopropylacrylamide). These hydrogels exhibit a hydrophilic swelling behavior at low temperatures (below their lower critical solution temperature, LCST), rapidly releasing cold-protectant substances such as salicylic acid, and a hydrophobic shrinking behavior at elevated temperatures, thereby slowing the release rate. This “switch” feature significantly enhanced the germination rate of coated corn seeds under cold stress, with an improvement of up to a 17.8%, and also boosted seedling physiological indices. Drawing inspiration from natural seed mucilage, Zvinavashe et al. [73] designed a dual-layer coating system: the inner layer, composed of silk fibroin/alginate, encapsulated plant growth-promoting rhizobacteria (PGPR), while the outer layer, made of pectin/carboxymethyl cellulose, provided physical protection. This innovative coating successfully promoted nodulation in common bean plants grown in drought-prone sandy soil, thereby enhancing plant drought tolerance. Athanasiou et al. [74] developed a calcium alginate/melatonin coating that effectively mitigated oxidative damage in tomato seedlings subjected to salt stress, demonstrating the potential of hydrogels as carriers for phytohormones to alleviate abiotic stress.

Despite promising laboratory results, the transition to field-scale application of hydrogel seed coatings faces significant practical hurdles. A primary concern is the mechanical integrity of the hydrogel coating during mechanized sowing operations. The abrasive forces and impacts experienced within seeders can cause coating fragmentation, delamination, or complete detachment from the seed surface [75,76]. This mechanical stress compromises the coating’s core functions of water retention and controlled release, and the dislodged gel particles may even clog precision seeding equipment. Therefore, the future design of seed-coating hydrogels must prioritize not only biological functions but also mechanical durability and adhesion strength to endure handling and sowing stresses.

### 3.3. Hydrogels for Controlled Release of Nutrients and Agrochemicals

Owing to their three-dimensional network structure, hydrogels serve as efficient carriers for the controlled release of nutrients and agrochemicals. The primary release mechanisms include physical encapsulation, governed by diffusion- and swelling-controlled processes, and chemical interactions, such as covalent or ionic bonding that enable sustained release. Release kinetics can be finely tuned by adjusting hydrogel hydrophilicity, crosslinking density, and environmental responsiveness, thereby improving nutrient and pesticide use efficiency while substantially reducing environmental pollution caused by leaching and runoff.

Conventional chemical fertilizers are prone to rapid dissolution, leading to nutrient loss and environmental contamination. Hydrogels with slow-release designs effectively address these limitations. Priya et al. [77] synthesized a cellulose nanofiber (CNF)/carboxymethyl cellulose (CMC) urea-loaded hydrogel that exhibited a water absorption capacity of 147 g/g and achieved 80% biodegradability within 30 days, while also promoting seed germination. Bora et al. [78] prepared a starch/itaconic acid composite hydrogel capable of triple controlled release of N, P, and K, with cumulative release rates of 98%, 81%, and 95% release over 20 days, respectively), significantly improving okra germination. Chiam et al. [79] developed a carboxymethyl cellulose/microfibrillated cellulose (CMC/MFC) hydrogel that degraded by more than 95% in soil within 60 days and enabled uniform NPK release through a calcium carbonate templating strategy; a 1 g application rate was found to optimally promote garlic chive growth. To cope with extreme environmental conditions such as those encountered in desert regions (high temperature and high salinity), smart responsive hydrogels offer distinct advantages. Zhang et al. [80] developed a temperature- and pH-dual-responsive core–shell hydrogel that retained 163.89% of its original water absorption capacity after 16 wet–dry cycles in a simulated desert environment. This material enabled gradient urea release for up to 40 days and achieved a 70% germination rate in wormwood. Wu et al. [81] developed a neutral-environment-responsive cellulose/chitosan hydrogel that exhibited optimal water retention performance and the slowest nutrient release rate among the tested systems.

**Figure 6:**
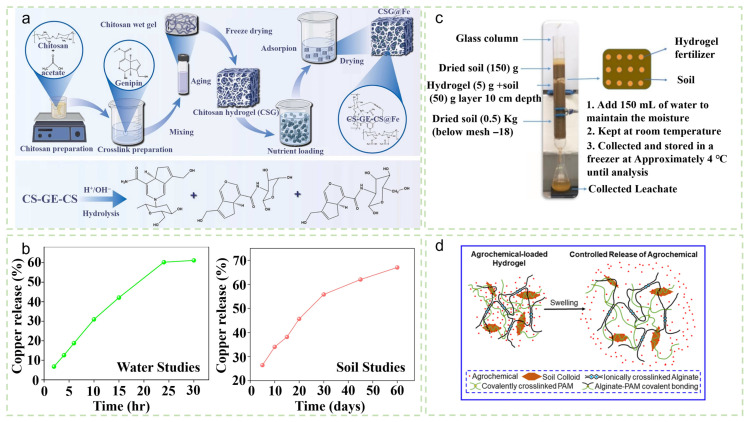
(**a**) Schematic of pH-responsive iron fertilizer synthesis and soil degradation principle. Reproduced with permission from reference [82]. Copyright 2024, Elsevier. (**b**) Copper release curves of pectin/poly(sodium methacrylate) hydrogel (PSMA/CMTKG) in water and soil. Reproduced with permission from reference [83]. Copyright 2025, Elsevier. (**c**) Simulated operational soil release test. Reproduced with permission from reference [86]. Copyright 2024, Elsevier. (**d**) Schematic of agrochemical slow release. Reproduced with permission from reference [85]. Copyright 2020, Elsevier.

Beyond macronutrient delivery, hydrogels are also effective platforms for the precise supplementation of micronutrients. Addressing the tendency of iron to oxidize and become fixed in soil, Liu et al. [82] developed a chitosan-based hydrogel (GE-CSG@Fe) with a high iron loading capacity of 326 mg/g. Its acid-responsive release behavior simultaneously regulated rhizosphere pH, preserved Fe^2+^ activity, and effectively promoted wheat and tomato growth (mechanism illustrated in Figure 6a). For copper supplementation, Kumari et al. [83] fabricated a pectin/poly(sodium methacrylate) hydrogel that sustained copper release for 60 days (67.02% release, Figure 6b), without exhibiting toxicity toward soil nitrogen-fixing bacteria. Hydrogels also demonstrate strong potential in pesticide-controlled release. Sun et al. [84] prepared a sodium carboxymethyl cellulose/quaternized chitosan/azadirachtin (CMC-Na/HACC/MT) hydrogel that exhibited excellent water retention (swelling ratio > 20 g/g) and enabled the sustained release of azadirachtin over 21 days, resulting in enhanced germination (germination rate increased to 75%) and antibacterial activity. Zhang et al. [85] utilized natural soil colloids as green modifiers to fabricate an alginate/polyacrylamide (PAM) hydrogel, which effectively delayed the release of both pesticide and fertilizer (34.4% pesticide release in 54 h and 56.9% fertilizer release in 30.5 h; schematic shown in Figure 6d).

The integration of nanotechnology further enhances the performance of hydrogel-based carriers. Sattar et al. [86] developed a latex/pectin hydrogel capable of stably loading and slowly releasing nano-fertilizers (NFs). In simulated soil release experiments (Figure 6c), a swelling rate of 1410% resulted in a nutrient slow-release rate of 0.033%, and treated chili plants exhibited a 185% increase in plant height. Gavriely et al. [87] employed a natural jellyfish-derived hydrogel to control the release of copper (I) oxide nanoparticles. The release kinetics closely matched crop nutrient demand, and the material was fully biodegradable after use, while simultaneously supplementing soil with nutrients such as sulfur and nitrogen. To address the non-degradability and potential secondary pollution associated with conventional petroleum-based hydrogels, the development of green and biodegradable materials has emerged as a major research focus. He et al. [88] developed a lignin-based hydrogel that retained a high water absorption capacity of 88.95 g/g after 8 cycles across a pH range of 4–10, while increasing soil water retention by 42.66% and total nitrogen storage by 110% and extending the release duration of urea and potassium ions by 48-fold and 60-fold, respectively. Ribeiro et al. [89] synthesized a sugarcane bagasse cellulose-based hydrogel free of epichlorohydrin residues, which showed no adverse effects on soil microbial communities and performed comparably to commercial products in promoting maize growth.

The stability of payloads in hydrogels is a frequently overlooked practical constraint. While dry hydrogels typically provide a good shelf life for stable agrochemicals, hydrolysis-sensitive compounds may degrade due to trapped residual moisture [90]. Upon application, UV radiation can degrade both the matrix and the payload, while oxygen and free radicals within the swollen gel may oxidize sensitive compounds. Additionally, competitive displacement by soil ions or organic matter can also lead to unexpected burst releases of nutrients or pesticides. Therefore, future research must prioritize the standardized evaluation of storage stability under realistic conditions and the development of accelerated aging tests to predict shelf life [91].

To clearly illustrate the water retention and holding properties of hydrogels, Table 2 summarizes their maximum water absorption capacity. This parameter is a key performance metric for hydrogels. It is fundamental to enhancing soil hydrological properties and, consequently, influences other physical and chemical parameters.

### 3.4. Hydrogels for Controlled Release of Microorganisms

**Figure 7:**
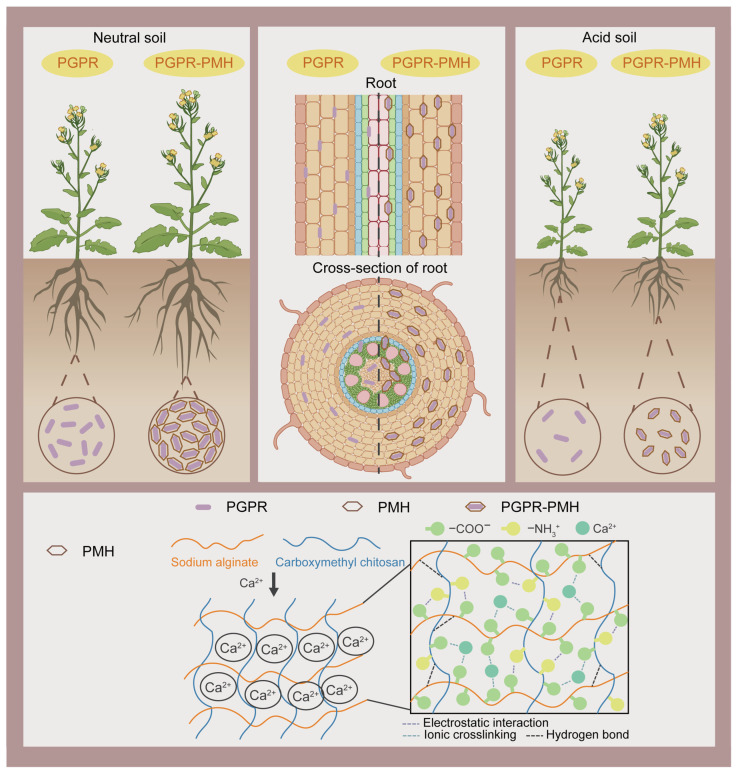
Schematic illustration of the ability of PMH to preserve, deliver, and promote PGPR colonization for plant growth and resistance in acidic environments. Reproduced with permission from reference [92]. Copyright 2025, Springer Nature.

Encapsulating microorganisms within hydrogels provides physical protection and a favorable microenvironment, effectively addressing challenges associated with direct microbial application, such as low cell survival, poor colonization, and environmental dispersion. Common encapsulation strategies include adsorption, covalent binding, and gel entrapment. The incorporation of biocompatible additives, such as glycerol, can enhance the mechanical strength of hydrogels without compromising microbial viability. The porous network structure of hydrogels facilitates efficient mass transfer, while their swelling behavior enables responsiveness to environmental stimuli, thereby regulating microbial release and functional expression. The delivery of plant growth-promoting rhizobacteria (PGPR) via hydrogels represents an important strategy to reduce reliance on chemical fertilizers. Feng et al. [92] developed a carboxymethyl chitosan/calcium alginate hydrogel (PMH) capable of effectively encapsulating the endophytic PGPR strain *Ensifer* C5 and promoting its targeted colonization in rapeseed roots. By modulating endodermal suberin deposition and auxin distribution in roots (mechanism schematic shown in Figure 7), this hydrogel system increased rapeseed field yield by 30% and enhanced plant stress resistance and disease tolerance through the upregulation of arachidonic acid metabolism.

In the context of environmental remediation, hydrogels used as microbial immobilization carriers significantly enhance microbial activity and operational stability. González-Morales et al. [93] immobilized *Aspergillus* sp. and *Rhodotorula* sp. within alginate hydrogels for the bioremediation of selenium-contaminated environments. The immobilized fungal system efficiently reduced toxic Se (IV) to inert Se (0) nanospheres, achieving a removal efficiency exceeding 43%, while confining the reduced selenium within the hydrogel matrix and thereby preventing secondary pollution. Zhang et al. [94] developed a polyvinyl alcohol/silica composite hydrogel in which an interconnected macroporous structure, formed at SiO_2_ contents above 2 wt%, markedly enhanced mass transfer efficiency and microbial metabolic flux. As a result, the system exhibited higher chemical oxygen demand (COD) removal efficiency and oxygen utilization rates in wastewater treatment applications. Conventional microbial immobilization methods, such as PVA-boric acid crosslinking, may adversely affect microbial viability. To overcome this limitation, Takei et al. [95] innovatively employed sodium sulfate as a crosslinking inducer to fabricate PVA hydrogel microspheres, thereby avoiding the toxicity associated with boric acid toward yeast cells. The resulting PVA-sodium sulfate microspheres exhibited improved water stability, and the encapsulated yeast cells demonstrated 2–3 times higher metabolic activity in ethanol fermentation compared with traditional PVA-boric acid microspheres. Moreover, the system maintained stable performance over at least 10 reuse cycles, highlighting its superior biocompatibility and process stability.

A critical challenge limiting the commercialization of microbial-loaded hydrogels is their limited shelf life and storage stability. The viability and metabolic activity of encapsulated microorganisms can decline rapidly during storage due to desiccation, nutrient depletion, or the accumulation of metabolic wastes within the hydrogel matrix. Strategies to overcome this challenge include (1) formulation optimization using protective colloids [96]; (2) employing mild drying techniques to produce “dry” inoculant hydrogels that are reactivated upon rehydration [97]; and (3) designing stimuli-responsive hydrogels that only become permeable or degrade upon contact with root exudates, thereby initiating microbial release and activity in situ. Therefore, future research must prioritize the standardized evaluation of storage stability under realistic conditions and the development of accelerated aging tests to predict shelf life [98].

Beyond shelf life, the ecological safety and effectiveness of hydrogel-based microbial delivery rely on two underexplored factors: (1) demonstrated rhizosphere colonization and function in situ. While most studies confirm survival after encapsulation, they often lack compelling evidence of sustained colonization and activity in the competitive rhizosphere. Moving beyond inference requires advanced methods—such as tracking GFP-labeled bacteria or using strain-specific qPCR [99]. (2) The assessment of unintended impacts on the soil microbiome is crucial. The introduction of any exogenous agent carries ecological risks. Both the hydrogel material and the released microorganisms can alter indigenous microbial communities, potentially reducing diversity or disrupting beneficial ecological networks [100,101]. This disruption could compromise soil multifunctionality or even promote pathogen emergence [102]. Moreover, the introduced strain could become a “biological pollutant” if it outcompetes native consortia or leads to unforeseen long-term effects.

### 3.5. Soilless Cultivation Technology

Conventional soil-based cultivation faces persistent challenges, including disease transmission, high resource consumption, and difficulty in achieving precise large-scale control, which have driven increasing interest in soilless cultivation technologies. However, hydroponic systems often provide insufficient mechanical support for plant roots, while commonly used inert substrates such as rockwool and coco coir typically suffer from limited water and nutrient retention, as well as relatively high costs. Owing to their exceptional water absorption and retention capacity, tunable physicochemical properties, and good biocompatibility, hydrogels have emerged as promising materials for the development of novel, high-performance substrates for soilless cultivation.

**Figure 8:**
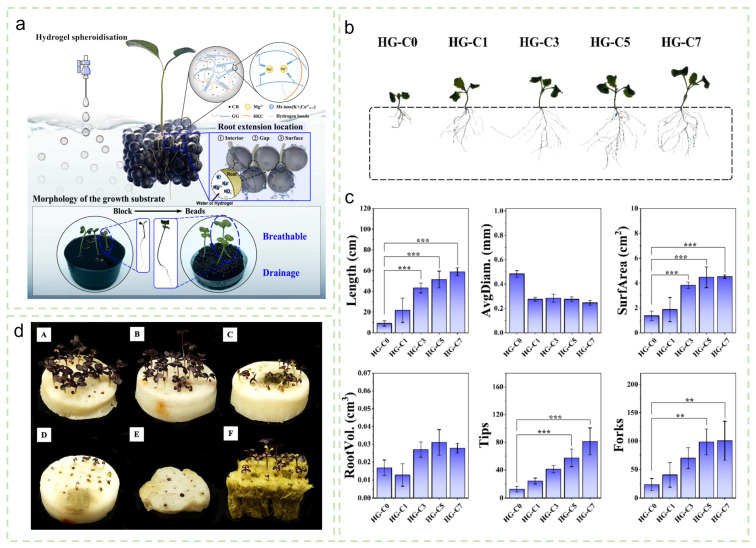
(**a**) Process of hydrogel spheronization, planting effect in stacked hydrogel bead growth substrate, types of root extension in the substrate (including nutrient uptake from hydrogel, absorption from interstitial water storage, and nutrient absorption at the gel-water storage interface), and images of rapeseed seedlings growing in bulk and spherical stacked substrates. (**b**) High-resolution images of rapeseed roots were scanned with a flatbed scanner after 21 days of continuous growth in HG-C0, HG-C1, HG-C3, HG-C5, and HG-C7. (**c**) Analysis of root parameters (total length, avg. diameter, surface area, root volume, tips, and forks) based on scanned images for rapeseed grown in different HG-C hydrogel substrates. Reproduced with permission from reference [104]. Copyright 2025, Elsevier. ** *p* < 0.01, *** *p* < 0.001 (**d**) Photographs of microgreens grown on substrates on day 12. From A to F: SF2, SF5, RF2, RF5, CT, and rockwool. Reproduced with permission from reference [107]. Copyright 2025, Elsevier.

The performance of hydrogel substrates is strongly dependent on their network structure. Qin et al. [103] optimized the crosslinker (MBA) content to fabricate a poly(acrylamide-co-isopropylacrylamide)/gelatin composite hydrogel. At an MBA concentration of 0.05%, the hydrogel achieved an optimal balance among mechanical strength, pore structure, and transparency. This formulation not only provided a stable supply of water and nutrient for soybean seedlings and promoted both root and shoot growth, but also enabled in situ observation of root dynamics due to its transparency. Dai et al. [104] further functionalized spherical hydroxyethyl cellulose hydrogel beads (HG-C) by incorporating carbon black (CB) and constructing a stacked cultivation system (Figure 8a). The incorporation of CB improved pore connectivity and enhanced light and thermal regulation within the substrate, significantly promoting rapeseed root growth under stress conditions such as low temperature (Figure 8b,c), with root length increasing by up to 648%.

Hydrogel substrates also exhibit clear advantages in resource-efficient utilization. Segura et al. [105] demonstrated that incorporating potassium polyacrylate hydrogel into hydroponic substrates significantly extended irrigation intervals, thereby reducing water and nutrient solution consumption. Life cycle assessment (LCA) further indicated a substantially lower environmental footprint for hydrogel-containing systems. In addition, hydrogel substrates can influence the uptake and distribution of specific substances by plants. Tadevosyan et al. [106] reported that addition of polymer hydrogel to a calcium-containing substrate altered the uptake and internal distribution patterns of radioactive nuclides ^90^Sr and ^137^Cs in chili plants, highlighting the potential of hydrogel substrates for controlled cultivation in contaminated environments. The development of environmentally friendly and biodegradable substrates represents an important future direction for soilless cultivation. Evensen et al. [107] employed freezing, freeze-drying, and post-crosslinking techniques to impart an interconnected macroporous structure to alginate hydrogels. The resulting substrate maintained excellent water retention while providing adequate air permeability, thereby creating a favorable environment for root respiration. In cultivation experiments, cruciferous microgreens grown on this alginate-based substrate exhibited higher germination rates and greater fresh weight compared with those cultivated on traditional rockwool (Figure 8d). This finding demonstrates the strong potential of alginate-based substrates as a sustainable alternative to non-degradable substrates.

To provide a clearer overview, Table 3 summarizes the main categories of agriculture-related hydrogels based on agrochemicals, mechanisms, and challenges.

### 3.6. Contextual Dependence and Performance Boundaries of Hydrogel Efficacy

While the preceding sections have detailed the significant potential of hydrogels to improve soil properties and crop performance, it is crucial to emphasize that these benefits are not universally guaranteed. The efficacy of hydrogel applications is highly context-dependent, governed by a complex interplay of material properties, environmental conditions, and management practices. Recognizing these performance boundaries is essential for setting realistic expectations and guiding the targeted and efficient use of hydrogel technology in agriculture.

(1) Boundaries Defined by Soil Texture and Initial Conditions

The efficacy of hydrogels in amending soil physical structure is not universal. Their impact is most pronounced in loose, macroporous sandy soils, where they primarily enhance water retention and provide cohesion. In fine-textured clay soils, however, the limited space for expansion constrains their ability to significantly increase total porosity. In such contexts, their primary value may shift towards regulating soil water potential and improving aeration, rather than substantially reducing bulk density (see Section 2.1). Furthermore, the initial soil pH, salinity, and organic matter content can profoundly influence hydrogel swelling behavior, nutrient release kinetics, and even structural integrity.

(2) Boundaries Imposed by Climate and Hydrological Regimes

The water-saving benefits of hydrogels are highly contingent upon local rainfall patterns and irrigation practices. Their buffering capacity is most valuable in regions characterized by seasonal drought or under intermittent irrigation schedules. In areas with frequent precipitation or high water tables, their utility diminishes, and they may even impede soil gas exchange persistently saturated conditions [108]. Additionally, intense ultraviolet radiation and significant temperature fluctuations can accelerate the photo- and thermal-degradation of certain natural polymer-based hydrogels, thereby shortening their functional lifespan in the field [109].

(3) Boundaries Related to Crop Species and Growth Stages

The agronomic response to hydrogels varies significantly with crop type and phenological stage. Shallow-rooted vegetables or horticultural crops, which exhibit high sensitivity to topsoil moisture during seedling establishment, often show more immediate benefits from hydrogels applied via seed coating or shallow incorporation [110]. In contrast, for deep-rooted field crops that primarily utilize subsoil water during mid-to-late growth stages, the overall yield enhancement attributable to hydrogels may be less pronounced than their positive effects on seedling survival and early vegetative growth [111].

(4) Boundaries of Temporal Dynamics and Long-Term Effects

The performance of hydrogels is dynamic and evolves temporally. As indicated in previous discussions (e.g., Section 3.2 and Section 3.4), hydrogel properties undergo progressive degradation through biological, chemical, and physical pathways. Consequently, key functional attributes such as swelling capacity, nutrient release kinetics, and structural support can diminish over multiple growing seasons [112]. This degradation necessitates periodic reapplication, which directly impacts long-term cost–benefit analyses and economic viability [113]. Furthermore, repeated or prolonged hydrogel application may alter soil microbial communities, organic matter dynamics, and hydraulic properties in ways that are not yet fully predictable. Both potential positive outcomes, including enhanced carbon sequestration, and possible negative repercussions highlight the critical need for sustained, field-scale monitoring.

(5) Boundaries of Degradation Process

The agricultural ideal of “biodegradability” requires materials to be fully assimilated by soil microorganisms into harmless byproducts, such as CO_2_, H_2_O, and biomass, within a timeframe aligned with crop cycles, leaving no persistent residues. Natural polymer-based hydrogels, including chitosan, starch, and cellulose, are generally considered biodegradable due to their enzymatically cleavable bonds [114]. However, their degradation rates vary widely: crosslinked starch or cellulose may degrade within weeks to months under active microbial conditions, whereas alginate or chitosan gels can persist longer, depending on acetylation or cross-linking density [115].

## 4. Conclusions and Future Perspectives

This review systematically consolidates the multidimensional applications of hydrogels in agriculture, spanning soil amendment, resource management, and crop productivity enhancement. Owing to their unique three-dimensional network architectures and highly tunable functional properties, hydrogels have demonstrated pronounced effectiveness in improving soil physical structure (e.g., reducing bulk density and optimizing porosity), enhancing water retention, regulating key chemical parameters (pH, electrical conductivity, and cation exchange capacity), and remediating contaminated soils. Concurrently, as intelligent carrier systems, hydrogels enable the precise and controlled release of water, nutrients, pesticides, and beneficial microorganisms, substantially improving resource use efficiency and exhibiting strong potential in advanced applications such as seed engineering and soilless cultivation. The diversity of hydrogel materials, including synthetic, natural-based, and composite systems, provides flexibility to address heterogeneous agricultural demands. Synthetic hydrogels (e.g., polyacrylamide-based) offer controllable performance and long-term structural stability but are often limited by dependence on non-renewable feedstocks, potential ecotoxicological risks, and slow degradation rates. In contrast, natural-polymer-based hydrogels (e.g., cellulose- and chitosan-derived) exhibit superior biocompatibility, biodegradability, and environmental friendliness, aligning well with the principles of green agriculture, though they commonly suffer from insufficient mechanical strength and environmental persistence. Composite hydrogel strategies that integrate natural and synthetic components with functional fillers (e.g., clay minerals and biochar) have emerged as a promising route to balance performance, environmental safety, and economic feasibility. Nevertheless, current studies still report divergent conclusions regarding several critical issues, including the long-term effects of hydrogels on soil saturated hydraulic conductivity, the mechanisms underlying the attenuation of water retention capacity under repeated wet–dry cycles, and the potential inhibitory effects of excessive application rates on early crop growth. Moreover, the biodegradation pathways and environmental fate of hydrogels remain insufficiently understood, underscoring the need for long-term, field-scale monitoring.

To fully unlock the potential of hydrogels in advancing sustainable agriculture, future research should prioritize breakthroughs in the following key directions: (i) Development of eco-friendly materials, including the development of scalable bio-waste-derived hydrogels, optimization of low-toxicity crosslinking strategies (e.g., enzymatic or radiation-induced), and assurance of full life-cycle environmental safety; (ii) Deepening environmental interaction mechanisms by quantifying osmotic stress effects (e.g., sodium ion dynamics), elucidating soil microbiome responses, establishing hydrogel degradation kinetics, and clarifying long-term impacts on soil hydraulic properties and nutrient cycling; (iii) Design of intelligent responsive systems through the development of multi-stimuli-responsive (pH, temperature, enzyme) hydrogels integrated with seed-coating technologies to enhance mechanical robustness and release precision; and (iv) Precision in application scenarios by constructing dose–response decision frameworks tailored to soil–crop systems and expanding integrated applications in saline–alkali land remediation, soilless cultivation, and extreme-environment agriculture.

In summary, as a transformative class of agricultural functional materials, hydrogels are poised to play an increasingly critical role in addressing global challenges related to water scarcity, soil degradation, and food security. Through sustained interdisciplinary innovation, rigorous environmental risk assessment, and holistic life-cycle management, hydrogel technologies hold strong promise to become a core driver of agricultural transformation toward higher resource efficiency, ecological intelligence, and systemic sustainability. Future research and practical deployment must advance in parallel, pursuing both performance optimization and ecological safety to ultimately achieve the synergistic goals of soil health improvement, stable crop productivity, and environmental conservation.

## Figures and Tables

**Table 1:** Classification and characteristics of typical hydrogels for agricultural applications.

Category	Typical Examples	Cross-Linking Method	Key Functional Groups	Primary Agricultural Application and Notes
Synthetic	Polyacrylamide (PAM), Poly (acrylic acid) (PAA), Potassium polyacrylate	Chemical (covalent)	–CONH_2_, –COO–K^+^	High WAC, slow degradation, concerns about ecotoxicity; used as water retainers.
Natural Polymer-based	Chitosan, Alginate, Cellulose derivatives (CMC), Starch	Physical (ionic, H-bonding) or Chemical	–OH, –NH_2_, –COO^−^	Biodegradable, biocompatible, often lower mechanical strength/WAC; used for nutrient/drug delivery.
Composite/Hybrid	PAM-grafted cellulose, Chitosan/Clay, Biochar-Hydrogel	Combination of physical and chemical	Multiple groups from components	Aim to balance performance (WAC, strength) and sustainability; emerging for smart delivery.

**Table 2:** Statistical table of water absorption capacity of hydrogel.

Hydrogel	WAC (g/g)	Ref.
CMTKG-PSA-PAM	189	[16]
POKgel	522	[18]
CMTKG-PSMA	248	[20]
GG-g-P(AA-AAm)/diatomite/DAP	115	[25]
FGB	115	[26]
CMC-H-B	3240	[27]
(CS + SL)@SA/AL	69	[28]
HES/CA33-PAM/sodium bentonite	1484	[29]
(AA-co-AAm)/WR	749	[30]
Locust bean gum/borax	130	[31]
LR-g-PAA/MMT/urea	15	[39]
MxTy-CH	204	[45]
CMC-g-poly(AM-AMPS)/Pal/UF	272	[47]
CS-CMC-SF	144	[51]
SA/urea	700	[54]
Alg:Ac	65	[58]
MS	200	[66]
galangal gum-based hydrogel	275.7	[67]
UCNF	147	[77]
starch/itaconic acid composite hydrogel	647	[78]
CMC/MFC	20	[79]
SAP3	188.74	[80]
CCH/PVA/NCC/NPK	79	[81]
Cu-loaded Pectin/PSMA/CMTKG	225.86	[83]
CMC-Na/HACC/FeCl3	20	[84]
alginate/polyacrylamide (PAM) hydrogel	56.64	[85]
SA/SX/OLS	91.9	[88]
cellulose-based hydrogels	200	[89]

**Table 3:** Promising Hydrogel Classes for Specific Deliverables.

Agrochemical	Desired Release Profile	Optimal Hydrogel Class	Rationale	Key Challenges
Nutrients (N/P/K)	Slow, sustained, synchronized with uptake	Zwitterionic composites (e.g., chitosan-g-PAA/clay)	pH-responsive carboxyl/amine groups enable ion-exchange-driven release synchronized with root exudate acidity.	Nutrient leaching in sandy soils; competitive ion interference (Ca^2+^, Na^+^).
Pesticides	Protected, triggered, or long-term	Hydrophobically modified nanocellulose hydrogels	Hydrophobic pockets improve loading efficiency of non-polar pesticides.	UV degradation of pesticides; hysteresis in release under drought.
Microbial Inoculants	Protected, gradual release of viable cells	Soft, Macroporous Natural Hydrogels (e.g., Alginate, κ-Carrageenan)	Tunable mesh size protects microbes from shear stress while permitting nutrient diffusion.	Viability loss during freeze-drying.

## Data Availability

The original contributions presented in this study are included in the article. Further inquiries can be directed to the corresponding authors.
